# Indigenous knowledge for disaster mitigation and climate threats in Mentawai, Indonesia

**DOI:** 10.4102/jamba.v17i1.1877

**Published:** 2025-05-22

**Authors:** Yessy Markolinda, Sawirman Sawirman, Mery Ramadani, Fitri Yusya, Nadiyatul Husna, Fadilla Azmi, Rezi F. Surya, Rd Aldifa Taufiqurrahman, Mira Lilia D. Boru Panjaitan

**Affiliations:** 1 Department of Public Health, Faculty of Public Health, Universitas Andalas, Padang, Indonesia; 2 Department of Linguistics, Faculty of Humanities, Universitas Andalas, Padang, Indonesia

**Keywords:** disaster, climate change, indigenous knowledge, Mentawai, mitigation

## Abstract

Climate change and natural hazards such as floods, earthquakes and tsunamis pose a serious threat to coastal communities, including the Mentawai, West Sumatra, Indonesia. This research aims to explore and integrate the indigenous knowledge of Mentawai people in disaster mitigation and climate change adaptation. Using mixed methods research with a sequential explanatory model, quantitative data were collected from 106 respondents through a cross-sectional survey, while qualitative data were obtained through interviews with nine participants, including village officials, religious leaders and local residents. Results show that the majority of the community understands and practices indigenous knowledge such as reading natural signs, the traditional ritual *Tinapat* and the use of traditional *kentongan*. These practices help them prepare for and adapt to the impacts of earthquakes, tsunamis and coastal abrasion. However, the application of indigenous knowledge is still limited and is only done occasionally by most respondents. Climate change has a significant impact, such as increased coastal abrasion, decreased agricultural yields and changes in seasonal patterns, which affects people’s livelihoods. In addition, religion plays an important role in raising environmental awareness through rituals, prayers and sermons. Government support to integrate indigenous knowledge into mitigation policies is still considered to need improvement. Community expectations include improving education, environmental awareness and the provision of supporting facilities and technology. This research concludes that the synergies between indigenous knowledge, cross-sector collaboration and culture-based policy support can increase community resilience to disasters and climate change in a sustainable manner.

**Contribution:**

This research contributes by enriching the literature on the integration of indigenous knowledge in disaster mitigation and climate change adaptation, especially in coastal indigenous communities. The results are expected to be a reference in the development of sustainable culture-based policies, as well as supporting the improvement of community resilience to environmental threats through synergies among local traditions, education and technological support.

## Introduction

Areas along the coastline face increased risks from the impacts of climate change, such as rising sea levels and extreme weather, which can exacerbate natural hazards such as flooding and coastal erosion. The combined effects of sea level rise and tsunamis pose significant challenges for coastal fringe areas worldwide requiring comprehensive risk assessment and adaptation strategies (Gandini et al. 2021; Tursina et al. 2021). Researchers and policymakers recognise the importance of adopting holistic methodologies to assess climate change risks in urban settings and formulate sustainable development initiatives (Kumar et al. 2021; Tursina et al. 2021).

International recognition of risks posed by natural hazard events because of climate change and the increasing vulnerability of populations falling victim to disasters is growing (Gunasena, Weerasinghe & Piyadasa 2018). As concerns about the impacts of natural hazard events increase, researchers and practitioners are interested in identifying effective processes to build individual and community resilience (Sutton et al. 2021). One way to gain insights into systems that can enhance the resilience of local communities is to explore community habits in avoiding the worst impacts of natural hazards through the implementation of disaster mitigation strategies (Suarmika et al. 2022; Sutton et al. 2022). One example is Simeulue Island in Indonesia, 43 km from the epicentre of the earthquake that triggered the Indian Ocean tsunami in December 2004. Music was used to convey risk information about tsunamis into the Sense of Coherence (SOC) of the Simeulue community. Findings suggest that the Simeulue community applied comprehensible techniques to optimise disaster mitigation learning (Sutton et al. 2021).

The increasing frequency and severity of natural hazards by climate change pose significant challenges to coastal communities (Talukder et al. 2024). As extreme weather events cause the sea levels to rise, the need for effective disaster mitigation strategies becomes even more urgent (Ginige, Mendis & Thayaparan 2022; McCaughey et al. 2017). Integrating indigenous knowledge with scientific approaches to enhance community resilience and reduce disaster impacts shows a significant benefit for disaster mitigation. Indigenous knowledge accumulated over centuries through life experiences and interactions with the environment includes invaluable insights into local hazards, vulnerabilities and adaptation strategies (Hiwasaki et al. 2014).

Recent research has emphasised the importance of incorporating indigenous knowledge perspectives in disaster risk reduction efforts (Sakya et al. 2022; Setiawati et al. 2023). The process of establishing Tanjung Benoa, Bali, as a Tsunami Ready community, emphasised the dynamic interaction among local leaders, stakeholder engagement and capacity-building initiatives (Sakya et al. 2022). Similarly, previous research examining the impacts of climate change and anthropogenic pressures on the Bintan Islands, Indonesia, advocated for integrating indigenous knowledge into policies for sustainable development (Setiawati et al. 2023).

Indigenous knowledge offers valuable insights into cultural responses to climate change extremes. Studies in Bangladesh highlight indigenous peoples’ adaptation strategies rooted in inherent cultural beliefs and practices. By recognising and integrating indigenous approaches, policymakers and practitioners can develop more holistic and culturally sensitive disaster mitigation frameworks (Garai, Ku & Zhan 2022).

The development of systematic frameworks to facilitate the incorporation of indigenous perspectives into disaster mitigation practices is a key issue (Fahmi, Timms & Shepherd 2014). The process of integrating local and indigenous knowledge with science emphasises the need for collaborative efforts between communities, scientists and policymakers. The framework, based on observing, documenting and validating indigenous knowledge, offers a systematic approach to utilising both traditional strengths and scientific expertise (Hiwasaki et al. 2014).

Previous research comparing West Sumatra Province with Bali shows that some areas have similar levels of earthquake and tsunami hazard, but their preparation and handling vary. Although Bali is vulnerable to earthquakes and tsunamis, it is factually much better prepared to deal with such disasters than West Sumatra Province. The Mentawai Islands is one of the regencies in West Sumatra Province, located in the coastal area of the Indian Ocean, which is a vulnerable area to seismic activity and sea level rise because of climate change, especially in the islands such as Siberut, Sipora and Pagai. This region has a high vulnerability to natural hazards such as earthquakes and tsunamis as seen from the history of natural hazards in the Mentawai Islands. In 2010, a tsunami caused by a 7.2 magnitude earthquake had claimed the lives of people on Pagai and Sipora Islands (Putra et al. 2023). Bosua Village is one of the areas prone to high wave threats, located in South Sipora sub-district, with a coastline length of 29.98 km (BPS Mentawai Islands Regency 2024).

Mentawai Islands Regency, as a natural hazard-prone area, has its own understanding in dealing with natural hazards. Indigenous knowledge is not only related to the knowledge and understanding of humans and their environment but also to how humans act in the face of diverse natural resources. Disasters that occur in the Mentawai Islands region create a culture where people’s lives coexist with disaster phenomena, encouraging people to continue to adapt in order to survive frequent disasters (Putra et al. 2023).

Given the region’s vulnerability to natural hazards such as earthquakes and tsunamis, as well as the impacts of climate change, it is crucial to understand and adapt indigenous knowledge that has proven effective in addressing environmental challenges. In-depth research is needed to identify and apply this knowledge in disaster mitigation and climate change adaptation efforts to enhance the resilience and sustainability of local communities. Therefore, this study aims to answer several key questions: (1) How do the Mentawai Tribal Communities integrate indigenous knowledge into disaster mitigation practices? (2) What are the main adaptation strategies used by the Mentawai Tribal Community in response to climate change? and (3) How can indigenous knowledge be integrated into government disaster mitigation policies? In line with these research questions, the primary objectives of this study are to explore and integrate the indigenous knowledge of Mentawai people in disaster mitigation and climate change adaptation.

## Research methods and design

This research is a mixed method study with sequential explanatory model that combines quantitative and qualitative research in sequence. The quantitative phase employs a cross-sectional study design to analyse the distribution and frequency of demographic characteristics and assess the level of knowledge possessed by the Mentawai tribe regarding disaster mitigation and climate change threats. The sampling formula was obtained from *Lemeshow* for unknown population (Equation 1 and 2):


$$n=\frac{Z^{2}\cdot P\cdot (1-P)}{d^{2}}$$ [Eqn 1]

$$n=\frac{1.96^{2}x\;0.5\;x\;(1-0.5)}{0.10^{2}}=\frac{0.9604}{0.01}=96.04$$ [Eqn 2]

The minimum sample required was 96 respondents. To prevent drop out, 10% of the sample was added so that the total sample for this quantitative research was 106 respondents. Qualitative research was used to find out how the indigenous knowledge of the Mentawai people has helped them in disaster mitigation, especially related to the threats associated with climate change such as sea level rise and the indigenous knowledge practices and adaptation strategies used by the community in facing natural hazards and climate change. The phenomenology research approach was used and involved interacting with participants who experienced the events.

Quantitative data were analysed using descriptive statistics with a focus on frequency distribution and percentages. This analysis helped to describe the characteristics of respondents and the pattern of answers associated with each variable and question in the questionnaire. The data are presented in a tabular form to provide a clear visual appreciation of the distribution of age, gender, education level, occupation, as well as the level of knowledge and perception of respondents towards disaster mitigation and climate change.

Meanwhile, the qualitative data were analysed using a *thematic analysis* approach. The analysis process included transcription of interviews, manual coding of data, identification of key themes and triangulation of data from various sources. Emerging themes included indigenous knowledge in disaster mitigation, adaptation strategies to climate change, the role of religion and expectations of government support. This process was conducted to understand deep patterns of meaning and relationships between themes relevant to the research objectives.

### Ethical considerations

Ethical clearance to conduct this study was obtained from the Universitas Andalas Faculty of Public Health Research Ethics Commission (No. B/81/UN16.12.D/PT.01.00/2024).

## Results

### Quantitative results

Based on Table 1, the characteristics of the research respondents are divided into several categories. Of the 106 respondents studied, the majority were ≤ 43 years old (54.7%), while the rest were > 43 years old (45.3%). In terms of gender, there were more female respondents (52.8%) than male (47.2%).

**TABLE 1:** Characteristics of research respondents.

Respondent characteristics	Frequency	%
**Age (years)**		
≤ 43	58	54.7
> 43	48	45.3
**Gender**		
Male	50	47.2
Female	56	52.8
**Education**		
Elementary school	37	34.9
Junior high school	39	36.8
Senior high school	21	19.8
Higher education	9	8.5
**Occupation**		
Fisherman	11	10.4
Merchants	3	2.8
Pastor	3	2.8
Farmers	75	70.8
Homemaker	8	7.5
More	6	5.7

**Total**	**106**	**100.0**

In terms of education, most respondents had a junior high school education (36.8%), followed by primary school (34.9%), senior high school (19.8%) and university (8.5%). In terms of occupation, the majority of respondents worked as farmers (70.8%), while only 10.4% worked as fishermen, and a few others worked as traders, pastors and housewives.

Table 2 presents the frequency distribution of respondents’ knowledge of disasters and climate changes. In terms of how often respondents heard about weather changes, the majority of respondents (34.9%) reported hearing about it ‘sometimes’, followed by respondents who heard ‘very often’ and ‘often’ with the same percentage of 23.6%. Meanwhile, 17.9% of respondents stated that they ‘never’ heard about weather changes.

**TABLE 2:** Frequency distribution of knowledge towards disasters and climate change.

Question	Frequency	%
**1. How often do you hear about climate changes?**		
Sometimes	37	34.9
Very often	25	23.6
Often	25	23.6
Never	19	17.9
**2. What type of disaster occurs most frequently in your area?**		
Flooding	87	82.1
Earthquake	16	15.1
Tsunami	3	2.8
**3. Do you think weather changes have an effect on the increased frequency of natural hazards?**		
Influential	66	62.3
Very influential	37	34.9
Don’t know	3	2.8

In terms of the types of disasters that occurred most frequently in the respondents’ areas, flooding was the most common, with 82.1% of respondents reporting that flooding occurred frequently. A total of 15.1% of respondents reported earthquakes as a frequent disaster, while only 2.8% mentioned tsunamis.

Regarding the influence of weather changes on the frequency of natural hazards, the majority of respondents (62.3%) believe that weather changes ‘affect’ the increase in the frequency of natural hazards. A total of 34.9% even stated that the weather changes are ‘very influential’, while only 2.8% did not know or had no opinion.

Based on Table 3, the frequency distribution of indigenous knowledge in disaster mitigation shows that the majority of respondents (73.6%) were aware of customs or culture used to reduce disaster risk, while 26.4% of respondents did not know.

**TABLE 3:** Frequency distribution of indigenous knowledge in disaster mitigation.

Question	Frequency	%
**1. Are you aware of any of your customs or cultures that are used to reduce disaster risk?**		
No	28	26.4
Yes	78	73.6
**2. How often is the habit or culture applied in daily life?**		
Very often	10	9.4
Often	6	5.7
Sometimes	73	68.9
Never	17	16.0
**3. What kind of customs or cultures are used in dealing with disasters? (Choose the most appropriate one)**		
Customary or religious rituals	33	31.2
Traditional building structure	3	2.8
Signs of nature	61	57.5
More	9	8.5

In terms of the application of this habit or culture in daily life, 68.9% of respondents admitted that they only apply it sometimes, 5.7% apply it often, 9.4% apply it very often and 16.0% never apply it.

Regarding the types of customs or cultures used in dealing with disasters, 57.5% of respondents stated that the natural signs were the most commonly used customs. In addition, 31.2% of respondents chose customary or religious rituals, while traditional building structures were only chosen by 2.9% of respondents, and 8.5% chose other customs that were not listed in the options.

The results of this table indicate that although most respondents are aware of the existence of indigenous knowledge in disaster mitigation, its application in daily life remains limited. Natural signs are the most commonly used method to identify potential disasters. This suggests that the community still relies on traditional approaches as an initial form of mitigation.

Table 4 illustrates the frequency distribution of knowledge and effectiveness of indigenous knowledge in dealing with weather changes among respondents. Most respondents (67.9%) stated that they were aware of local customs or culture used to cope with climate changes, while 32.1% of respondents did not know. This shows that the majority of people still have knowledge of indigenous knowledge related to adjusting to climate change.

**TABLE 4:** Frequency distribution of indigenous knowledge in dealing with climate changes.

Question	Frequency	%
**1. Are you aware of any customs or cultures used to deal with climate changes?**		
No	34	32.1
Yes	72	67.9
**2. How effective are these habits or cultures in reducing the impact of climate change?**		
Highly effective	33	31.1
Effective	44	41.5
Less effective	23	21.7
Ineffective	6	5.7
**3. How do the people of Suku Mentawai adjust to climate changes? (Choose the most appropriate one)**		
Changes in cropping patterns	18	17.0
Natural resource management	45	42.4
Use of traditional technology	6	5.7
Customary rituals	6	5.7
More	31	29.2

When asked about the effectiveness of the habit or culture in reducing the impact of climate change, 41.5% of respondents rated it as ‘effective’, while 31.1% considered it ‘very effective’. A total of 21.7% rated the habit as ‘less effective’, and only 5.7% rated it as ‘not effective’. These results show that although some respondents doubted its effectiveness, most respondents felt that the indigenous knowledge was helpful in reducing the impact of climate changes.

In terms of how the Mentawai people adapt to climate changes, natural resource management is the most widely used method, chosen by 42.4% of respondents. A total of 17% of respondents mentioned changes in cropping patterns as a way of adaptation, while 5.7% chose the use of traditional technology and traditional rituals. A total of 29.2% chose the ‘other’ category, which may include various other adaptation methods not included in the available options.

The majority of respondents believe that the natural resource management is the primary approach to adapting to climate change. However, challenges remain in the utilisation of traditional technology and customary rituals as part of long-term mitigation. This highlights the need for a more holistic approach to integrating indigenous knowledge into climate change policies.

Table 5 shows community perceptions and expectations regarding the role of local customs or culture in reducing disaster risk and climate change. Most respondents, 48.1%, rated the role of such customs or culture as ‘very important’, and another 43.4% considered it ‘important’. Only a small proportion of respondents, 2.8%, felt this role was ‘less important’, while 5.7% considered it ‘not important’.

**TABLE 5:** Frequency distribution of perceptions and expectations.

Question	Frequency	%
**1. How important a role do you think habits or culture play in reducing disaster risk and climate change?**		
Very important	51	48.1
Important	46	43.4
Less important	3	2.8
Not important	6	5.7
**2. Do you feel the government supports the use of customs or culture in reducing disaster risk and climate change?**		
Very supportive	28	24.5
Support	58	54.7
Less favourable	16	15.1
Not in favour	6	5.7
**3. What are your expectations for the integration of customs or culture in reducing disaster risk and climate change?**		
Support from the government	43	40.6
Improved education and awareness	55	51.9
Provision of resources and technology	8	7.6

Regarding government support for the use of local customs or culture, more than half of the respondents (54.7%) felt that the government was ‘supportive’, and 24.5% considered the government to be ‘very supportive’. However, there were also 15.1% of respondents who rated the government as ‘less supportive’, and 5.7% felt that the government was ‘not supportive’.

For expectations related to the integration of local customs or culture in reducing disaster risk and climate change, the majority of respondents (51.9%) hoped for increased education and public awareness. A total of 40.6% of respondents expected the government to provide greater support, while another 7.69% expected the provision of more adequate resources and technology.

### Qualitative results

Based on Table 6, it can be seen that there were nine participants who were interviewed regarding the research topic, namely three village officials, three religious leaders or priests and three local residents. The aspects explored in the interviews are as follows:

**TABLE 6:** Characteristics of research participants.

Participants	Age (years)	Last education	Job or title
Participant 1	36	Bachelor’s degree	Bosua Village Secretary
Participant 2	42	Senior High School	Bosua Village Apparatus
Participant 3	32	Bachelor’s degree	Bosua Village Apparatus
Participant 4	50	Junior High School	Fishermen or local residents
Participant 5	40	Senior High School	Farmers or local residents
Participant 6	55	Junior High School	Farmers or local residents
Participant 7	40	Bachelor’s degree	Pastor
Participant 8	48	Bachelor’s degree	Pastor
Participant 9	35	Senior High School	Pastor

**TABLE 7:** Triangulation matrix – The role of religion in disaster mitigation and climate change.

Aspects	Pts-7	Pts-8	Pts-9	Conclusion
The role of religion in disaster mitigation	Religion teaches harmony with nature and responsibility for God’s creation.	Special prayers and rituals are performed to seek protection from natural hazards.	Sermons are used to educate the public about the importance of environmental conservation.	Religion acts as a motivator in environmental conservation and disaster mitigation.
Religious traditions and neighbourhoods	Religious teachings encourage people to protect forest and marine ecosystems as part of their faith.	Local beliefs involve nature as a major component in religious rituals.	The role of religious leaders as mediators to encourage the preservation of local ecosystems.	Religious traditions strengthen the motivation to conserve the environment.
Adaptation to climate change	Encourage people to reduce environmental damage based on religious moral teachings.	Religious leaders help facilitate climate change-related dialogue within communities.	Weather changes are considered a test of faith and require cross-sectoral cooperation.	The role of religion can be strengthened in climate change adaptation efforts at the community level.

Pts, participants.

#### Knowledge of disasters

Based on Table 8, the Mentawai People have an understanding of the types of disasters that often occur in their area. Disasters such as floods, earthquakes, coastal abrasion and tsunami are the main threats. This information is obtained through the observation of natural phenomena that they have learnt from generation to generation. For example, the community utilises traditional bells to quickly disseminate information related to disaster hazards. In addition, the Mentawai people have local knowledge to read the signs of a tsunami, such as by measuring the wave line as an early sign of a tsunami through observing sudden changes in seawater, such as the sudden receding of water to expose the seabed or the appearance of abnormally high waves, as well as measuring the line of wave impact on the beach. This knowledge is not only passed down orally by traditional leaders to the younger generation but also practised in everyday life as a preparedness effort.

**TABLE 8:** Triangulation matrix of sources of knowledge about disasters.

Aspects	Pts-1	Pts-2	Pts-3	Pts-4	Pts-5	Pts-6	Conclusion
Type of disaster	Flood and earthquake	Flood, earthquake and coastal abrasion	Flooding and coastal abrasion	Flooding	Flooding and coastal abrasion	Floods and tsunamis	Floods and earthquakes are the main types of disasters that occur most frequently in the region.
How to recognise a disaster?	Using traditional bells for communication.	Relying on signs of nature such as waves splitting or fire in the sea.	Direct observation of earthquakes and extreme weather changes.	Using animal sounds and ocean waves as indicators.	Understanding natural signs such as large waves as a sign of a tsunami.	Paying attention to wave phenomena and wild animal sounds.	Local knowledge such as bells and natural signs are the main ways of recognising disasters.

Pts, participants.

#### Traditions in the face of disaster

Based on Table 9, indigenous knowledge plays an important role in helping communities face and mitigate disasters. One important tradition is the *Tinapat* ritual, which consists of special prayers to ask for protection from ancestors and prohibitions against consuming certain animals, such as turtles. This ritual is believed to maintain the balance between humans and nature, strengthening community resilience by fostering collective awareness, preparedness and adherence to traditional environmental practices that help reduce disaster risk. In addition, the community also has a tradition of holding harvest celebrations, which not only serve as an expression of gratitude but also as an opportunity to pray for protection from disasters. The custom of climbing hills during earthquakes is also an important mitigation measure, especially in response to tsunami threats. All these traditions have been passed down through stories, community practices and the guidance of traditional leaders, who continue to preserve their relevance in the face of modern challenges.

**TABLE 9:** Triangulation matrix of traditions in the face of disasters.

Aspects	Pts-1	Pts-2	Pts-3	Pts-4	Pts-5	Pts-6	Conclusion
Disaster-related traditions	*Tinapat* ritual to maintain the balance of nature.	Harvest feasts involve prayers and ceremonies to ask for protection.	Prohibition of consumption of certain animals such as turtles to prevent disasters.	Performing traditional rituals before and after a disaster.	Teaching the tradition of going uphill during an earthquake as a mitigation measure.	Protecting sea turtles so that the ecosystem is maintained as a form of nature protection.	Traditions such as traditional rituals, prohibitions on the consumption of certain animals and maintaining ecosystems are part of local disaster mitigation.
Ancestral heritage	Traditions are passed down from generation to generation from the ancestors.	Rituals have become an important part of local culture.	Traditions are told by traditional leaders to the younger generation.	Knowledge is passed on through indigenous community activities.	A hereditary habit of preserving nature.	This tradition is passed down from parents to the younger generation through stories.	Local traditions are passed down orally from generation to generation through indigenous communities and families.
Effectiveness of tradition	Effective in encouraging people to protect nature and ecosystems.	Traditions help communities stay alert to disasters.	Traditions are considered relevant for modern disaster mitigation.	Rituals give people a sense of security and trust.	Traditions such as climbing hills are effective to save themselves during a tsunami.	The habit of taking care of nature helps lower the risk of disasters.	Local traditions are still effective as early mitigation measures and maintaining harmony with nature.

Pts, participants.

#### Impacts of climate change

Based on Table 10, climate change has a significant impact to the Suku Mentawai community. These impacts include environmental damage because of coastal abrasion, decreased agricultural yields, reduced fish populations and changes in seasonal patterns that affect planting and harvesting schedules. In addition, education on the importance of maintaining marine ecosystems and reducing destructive activities, such as illegal logging, is increasingly encouraged. The role of village officials in supporting environmental rehabilitation programmes, in collaboration with non-governmental organisations (NGOs) and universities, is a key factor in raising community awareness of climate change adaptation, demonstrating a cross-sector collaboration to create sustainable adaptation strategies.

**TABLE 10:** Triangulation matrix of impacts of climate change.

Aspects	Pts-1	Pts-2	Pts-3	Pts-4	Pts-5	Pts-6	Conclusion
Climate change impacts	Illegal logging causes abrasion and mangrove destruction.	Extreme weather damages ecosystems and affects agricultural yields.	Decreased agricultural yields and reduced fish populations.	Seasonal patterns change and seafood yields drop dramatically.	Coastal abrasion increases, disrupting marine life.	Weather changes affect planting and harvesting patterns.	Climate change affects agricultural yields, fisheries and causes environmental damage.

Pts, participants.

#### The role of religion in disaster mitigation and climate change

Based on Table 7, religion plays an important role in shaping people’s views on environmental conservation and disaster mitigation. Religious teachings that emphasise harmony with nature motivate people to protect the ecosystem. Religious leaders use prayers, rituals and sermons as a means to educate communities on the importance of protecting the environment. In local beliefs, nature is often considered an integral part of spiritual life, so protecting ecosystems is a form of honouring the creator. In this case, religion not only provides moral reinforcement but also facilitates the implementation of community-based mitigation programmes that integrate local traditions.

#### Government’s role in disaster mitigation

Although the government has undertaken several disaster mitigation initiatives, the participation of local cultures is considered not optimal (Table 11). Existing mitigation programmes tend to involve less indigenous knowledge that has proven effective in dealing with disasters. However, cooperation with NGOs and universities for mitigation socialisation has had a positive impact in improving community preparedness. Village governments are expected to be more proactive in integrating local customs into mitigation policies, such as providing education on environmental conservation and training on culture-based mitigation skills. In addition, the community expects the government to provide facilities and tools to support mitigation measures, especially in the face of disasters such as floods and earthquakes. Local culture-based policies are seen to strengthen mitigation strategies that are more in line with community needs.

**TABLE 11:** Triangulation matrix – The role of government in disaster mitigation.

Aspects	Pts-1	Pts-2	Pts-3	Pts-4	Pts-5	Pts-6	Conclusion
Government’s role in disaster mitigation	Mitigation programmes have not optimally involved local culture.	There are no regulatory facilities or tradition-based policies.	Collaboration with NGOs and universities for disaster mitigation socialisation.	The government is expected to provide education and regulatory support for environmental conservation.	Collaboration with village officials in ecosystem rehabilitation activities is expected.	Expect policies that support environmental conservation from the government.	Village governments need to improve the integration of local customs in disaster mitigation policies.

Pts, participants.

## Discussion

### Quantitative

The characteristics of the respondents studied showed an almost even distribution of age and gender, and most respondents worked as farmers. In terms of knowledge, while most respondents heard about climate change, the frequency varied from frequent to infrequent. Flooding was the dominant natural hazard threat in the respondents’ areas, and most respondents were aware of the link between climate change and increased risk of natural hazards.

This result is in line with a study from Sloggy et al. (2021) who found that experiencing a natural hazard, such as a hurricane, significantly increased the likelihood of individuals believing that climate change is occurring (Sloggy et al. 2021). Other studies have also shown that climate change alters the frequency and intensity of climate-related disasters, thereby increasing disaster risk. This finding is in accordance with respondents’ beliefs regarding the significant impact of climate change on the frequency of natural hazards (United Nations Office for Disaster Risk Reduction n.d.).

Most respondents still have knowledge of local cultural practices related to disaster mitigation. Although most respondents are aware of local customs in disaster mitigation, they are rarely applied consistently in their daily lives. The results of this study indicate that people tend to rely on the observation of natural signs and traditional rituals as the main ways to mitigate disaster risks although only a few still utilise aspects such as traditional building structures.

In line with a study focused on indigenous village communities in West Java, it was revealed that these communities have significant indigenous knowledge related to disaster mitigation. This wisdom includes practices such as building houses that are resistant to natural hazards and maintaining sacred forests (Darmawan, Mulyana & Kurniawati 2022). Likewise, the Bedouin community in Banten, Indonesia, is an example of how indigenous knowledge plays an important role in disaster mitigation. Their traditional practices, which include rituals and natural signs, serve as important tools to manage disaster risk. This research emphasises the importance of integrating such indigenous knowledge into modern disaster management strategies, in line with the idea that respondents rely on traditional methods for mitigation (The Climate Reality Project 2016).

The Mentawai Tribal community relies on a diverse approach, with natural resource management as the main focus in dealing with climate change. In line with a study conducted by Darmawan et al. (2022) also on traditional villages in West Java, it was noted that natural resource management and changes in cropping patterns are key adaptation methods for communities. These practices, such as growing rice without tractors or chemical fertilisers, help break the cycle of leafhoppers and other pests, thereby reducing the risks associated with extreme weather events (Darmawan et al. 2022).

These results show that the majority of respondents consider local customs and culture to play an important role in reducing risks associated with climate change. However, there is still a view that support from the government is not optimal in the eyes of some respondents. This finding reflects respondents’ expectation for an active role of the government and increased community awareness in utilising indigenous knowledge as part of disaster mitigation and adaptation strategies to climate change.

Research from Ayuningtyas et al (2021) and Titko, Ristvej and Zamiar (2021) emphasises that indigenous knowledge is critical to adapting to climate change impacts. It suggests that the traditional practices not only help reduce risks but also increase community awareness and preparedness if supported by government initiatives. This is in line with respondents’ belief in the effectiveness of indigenous knowledge if backed by stronger institutional support (Ayuningtyas et al. 2021; Titko et al. 2021).

### Qualitative

The Mentawai people have a hereditary understanding of disasters such as floods, earthquakes, coastal erosion and tsunamis. They use traditional bells to disseminate hazard information and read tsunami signs, such as sudden changes in sea water or abnormal waves. This knowledge is passed down orally by traditional leaders and applied in daily life as part of preparedness efforts.

Previous research on communities on the slopes of Mount Merapi also found similar things, where *kentongan* is used as an important emergency communication tool. The *kentongan* is used to notify residents of an impending disaster, with specific rhythms indicating different levels of urgency. For example, a two-by-two cadence signals residents to be alert, while a strong cadence requires immediate evacuation (Hidayati, Widiarti & Nugroho 2022).

This study shows that the *Tinapat* ritual not only serves as a spiritual expression of the community but also holds disaster mitigation value by enhancing community preparedness. Additionally, customary regulations on the consumption of certain animals (e.g. the prohibition of consuming turtles) can be linked to efforts to protect coastal ecosystems, which contribute to long-term disaster mitigation. However, these practices are still largely regarded as part of cultural heritage rather than formally integrated strategies within disaster risk reduction policies.

A report by ICCROM emphasises that the indigenous and traditional knowledge is essential for disaster mitigation. Such practices have been shown to increase resilience to disasters, as traditional knowledge often includes effective methods for managing local hazards and utilising local materials for construction and preparedness (ICCROM 2024). A systematic review also highlighted that traditional practices not only provide immediate solutions but also encourage community involvement and empowerment in disaster mitigation efforts (Syuryansyah & Habibi 2024).

Climate change impacts coastal erosion, decreased agricultural yields, reduced fish populations and changes in seasonal patterns in Suku Mentawai. A study on coastal vulnerability in North Pagai Island highlighted that climate change is exacerbating the risk of coastal erosion and flooding. The study suggests that the rising sea levels and increasing storm intensity threaten the livelihoods of coastal communities, including the Mentawai people, who depend on fisheries and agriculture to make ends meet (Mutmainah & Putra 2017).

The implementation of climate adaptation strategies in the Mentawai Islands shows that the changing climate patterns are adversely affecting crop yields. Traditional agricultural practices are increasingly challenged by unpredictable rainfall and temperature changes, leading to decreased food security for the Mentawai people (Kusumaningrum, Cahyadi & Sitohang 2023).

Religious rituals, such as the *Tinapat*, are deeply intertwined with indigenous knowledge. While religious teachings shape moral and ethical perspectives on environmental conservation, indigenous knowledge offers practical guidance for disaster mitigation. Thus, religious practices in Mentawai are not separated from indigenous knowledge but rather serve as an integral part of it.

As shown in Table 7, religious teachings play a crucial role in shaping community awareness of the environment and disaster preparedness. Religious rituals and sermons from spiritual leaders serve as indirect educational tools that enhance the community’s understanding of the importance of maintaining ecological balance. However, the role of religion in mitigation is more long term and behaviour based, rather than a structural mitigation measure such as the development of early warning systems or safer spatial planning.

The study by Fiahenoo, Gedzi and Owusu (2024) highlights that the religious beliefs can foster a sense of responsibility towards nature, encouraging adherents to engage in sustainable practices as part of their spiritual duties. (Fiahenoo et al. 2024) The ICRS report (2024) notes that the religious leaders often utilise sermons, rituals and community events to raise awareness about environmental issues, thus motivating congregants to take action to protect their environment (Indonesia Consortium For Religious Studies 2024).

The government’s disaster mitigation programme is lacking indigenous knowledge although cooperation with NGOs and universities has improved community preparedness. Village governments are expected to proactively integrate local traditions into mitigation policies, provide education, offer culture-based training and support facilities. Local culture-based policies are considered capable of strengthening mitigation strategies according to community needs.

A report from Victoria (2016) notes that when communities are actively involved in assessing risks and planning mitigation strategies, the solutions developed are more relevant and effective. This approach corrects the shortcomings of top-down methods, which often fail to address local needs and capacities (Victoria 2016).

In Bali, the provincial government uses customary rules and gotong royong task forces to deal with disasters. This approach demonstrates how the application of local norms and regulations can streamline disaster management efforts, potentially reducing dependence on foreign aid and increasing community self-reliance (Kurniadi, Bahar & Savitri 2021).

The findings of this study highlight the importance of incorporating indigenous knowledge into more systematic disaster mitigation policies. The government and other stakeholders need to design community-based education programmes that teach disaster mitigation techniques based on the local wisdom. Furthermore, community involvement in policy planning at the local level is also key to enhancing the effectiveness of culturally based mitigation efforts.

This study has several limitations. Firstly, the qualitative sample size was relatively small (nine participants), which may limit the generalisability of the findings. Secondly, response bias could not be entirely ruled out, particularly in the subjective interpretation of the role of indigenous knowledge in disaster mitigation. Additionally, while the study identified key indigenous practices, it did not specifically quantify their impact on reducing disaster risks. Further research with a larger sample and a more comprehensive evaluation of the practical impact of indigenous knowledge on disaster preparedness is recommended.

## Conclusion

The results show that the Mentawai Tribal Community have customary knowledge that can be adapted in disaster mitigation and climate change. Tradition, local wisdom, the role of religion and cross-sector collaboration become important elements in creating a holistic mitigation strategy. This study recommends an increased synergy between government, academia and local communities to optimise the utilisation of local knowledge in disaster mitigation policies. The development of community-based training modules and increasing technological support to strengthen local wisdom are needed.
